# Transcriptomics as a predictor of biopharmaceutically favourable glycan profiles

**DOI:** 10.3389/fcell.2024.1504381

**Published:** 2024-12-17

**Authors:** Ben West, Pavlos Kotidis, Alena Istrate, Daniele Perna, Gary Finka, A. Jamie Wood, Daniel Ungar

**Affiliations:** ^1^ Departments of Biology, University of York, York, United Kingdom; ^2^ Biopharm Process Research, GlaxoSmithKline Research and Development, Stevenage, United Kingdom; ^3^ Departments of Mathematics, University of York, York, United Kingdom

**Keywords:** glycosylation, transcriptomics, monoclonal antibodies, cell line development, biopharmaceutical production

## Abstract

*N*-glycosylation plays a crucial role in defining the pharmacological properties and efficacy of therapeutic proteins, commonly referred to as biologics. The inherent complexity and lack of a templated process in glycosylation leads to a wide variation in glycan structures, posing significant challenges in achieving consistent glycan profiles on biologics. This study leverages omics technologies to predict which cell lines are likely to yield optimal glycosylation profiles, based on the existing knowledge of the functional impact of specific glycan structures on the pharmacokinetics, immunogenicity, and stability of therapeutic antibodies. The study highlights that bulk RNA-sequencing data holds predictive power for glycosylation outcomes in of monoclonal antibodies (mAbs). For instance, Alg5 is identified to be predictive, before beginning a mAb production run, of mAbs bearing higher levels of Man5. This is inferred to increase glycosylation site occupancy on endogenous proteins, thereby intensifying competition for glycosylation enzymes in the Golgi and indirectly influencing mAb glycan processing. Additionally, the elevation of the UDP-Gal transporter in cell lines expressing mAbs with a single galactose residue is also observed intranscriptomic data prior to beginning a production run. These findings suggest that early-stage transcriptomics can aid in the streamlined development of cell lines by enabling pre-emptive adjustments to enhance glycosylation. The study also underscores that while transcriptomic data can predict certain glycosylation trends, more crucial factors affecting glycan profiles, such as enzyme localization within the Golgi apparatus and endogenous competition for glycosylation machinery, are not captured within the transcriptomic data. These findings suggest that while transcriptomics provides valuable insights, enzyme localization and intracellular dynamics are critical determinants of glycosylation outcomes. Our study starts to address the relevant mechanisms essential for improving cell line development strategies and achieving consistent glycosylation in biologics production.

## Introduction

Cell line development (CLD) is a pivotal step in biopharmaceutical manufacturing, focused on developing and identifying a single, high-producing clone from a large pool of candidates. This process is essential for generating stable cell lines that consistently express therapeutic monoclonal antibodies (mAbs) at high levels, meeting all necessary quality and regulatory standards. The selection and optimisation of the cell line involves rigorous screening, process development, and scaling to ensure production of clinical grade material suitable for therapeutic use (Munro et al., 2017). One aspect of the screening process is ensuring that critical quality attributes of the mAbs, such as glycosylation status, meet stringent criteria (Sha et al., 2016).

**FIGURE 1:**
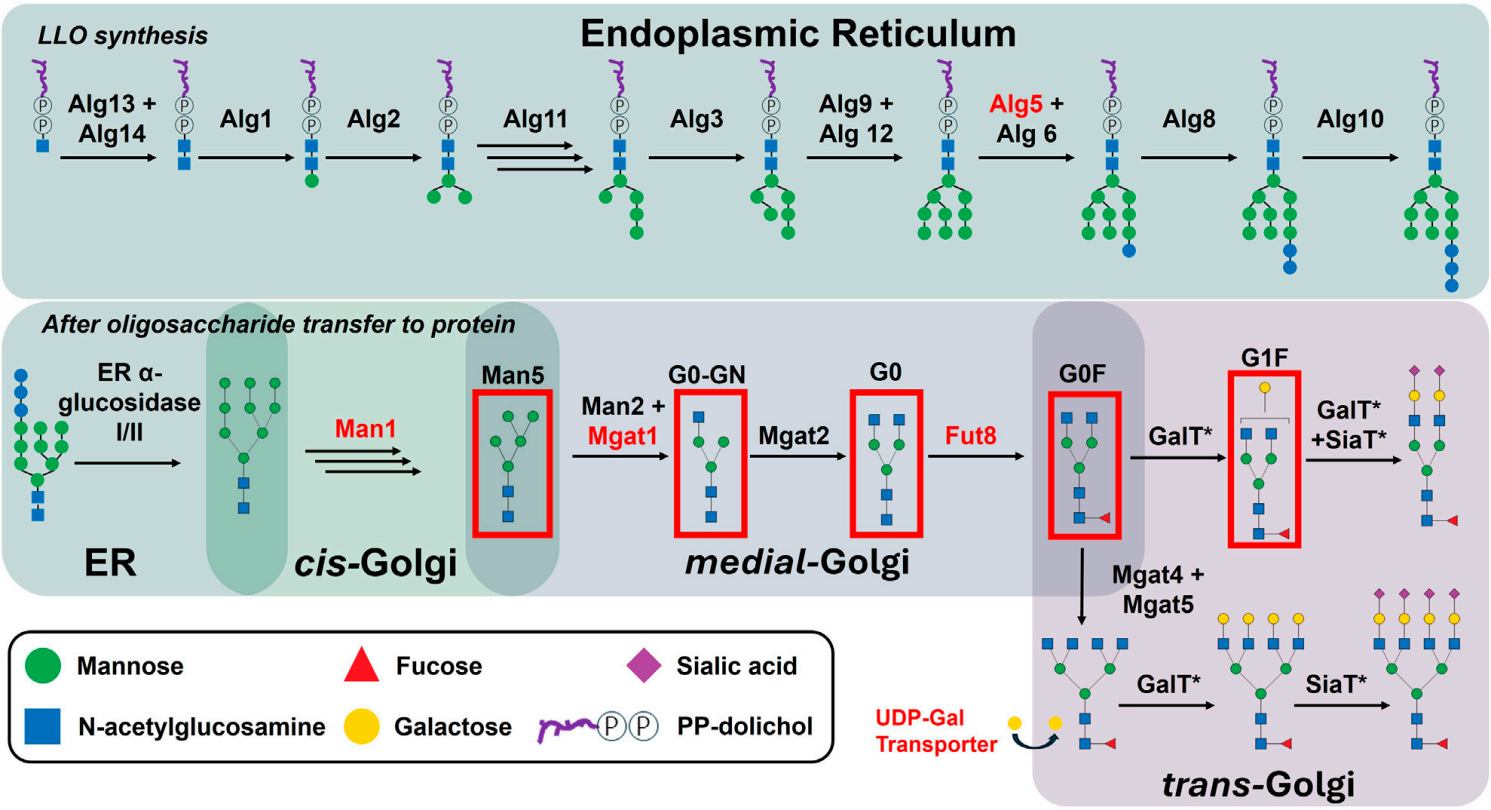
Glycan processing during lipid-linked oligosaccharide synthesis and post transfer to protein. Enzyme abbreviations: mannosidase I (Man1), mannosidase II (Man2), fucosyltransferase 8 (Fut88), N-acetylglucosaminyltransferases I-V (Mgat1-5), galactosyltransferases (GalT), sialyltransferases (SiaT), lipid-linked oligosaccharide (LLO). The glycans which will be discussed in this work have been boxed in red and their used nomenclature indicated. Of note: Key enzymes for this work are highlighted in red, while those marked with an asterisk also participate in O-glycan and glycolipid processing, competing for enzyme activity.

Glycosylation is a crucial post-translational modification of IgG antibodies produced by mammalian cells, such as the Chinese hamster ovary (CHO) cell lines commonly used in biopharmaceutical production (Li et al., 2010). Specifically, IgG_1_ molecules contain a single conserved *N-*linked glycan at Asn297 decorating each of the two heavy chains. During *N-*glycan synthesis, various sugar moieties can be added, resulting in the formation of varied glycan structures, such as Man5, G0-GN, G0, G0F, G1F among others (Figure 1).

The co-existence of these structures in the final IgG preparation yields a heterogenous mixture of glycoforms, each of which can differently impact the efficacy, stability, and immunogenicity of mAbs. They do this by modulating the binding affinity of the IgG to Fcγ receptors which in turn influences different antibody related functions including complement-dependent cytotoxicity (CDC) and antibody-dependent cell-mediated cytotoxicity (ADCC) (Jefferis, 2009). Glycoform distributions during mAb production can be influenced by both glycosylation engineering and cell culture conditions (Serrato et al., 2007; Nettleship et al., 2009; Sou et al., 2017). Importantly, glycan structures attached to the mAbs are determined by the expression and arrangement of glycosylation machinery components within the host cell line (Fisher et al., 2019b). Controlling glycosylation during CLD is therefore essential, as particular glycoforms may be necessary to achieve optimal therapeutic efficacy whilst other glycoforms may need to be minimized or eliminated to ensure drug safety.

Known control mechanisms such as the expression, regulation, and intracellular localisation of *N*-glycosylation enzymes and sugar nucleotide transporters, play a significant role in shaping the glycan profiles observed in mAbs. A summary of *N-*glycosylation enzymes relevant for this study can be found in Supplementary Table 1. *N*-glycosylation, in particular, is initiated in the cytosol, with precursor glycan structures assembled onto the ER membrane before being transferred to the nascent protein in the lumen of the endoplasmic reticulum (ER). Subsequently, glycosylation enzymes, such as glycosyltransferases and glycosidases, are responsible for the sequential trimming and addition of sugar moieties as the glycan structures traverse the ER and Golgi apparatus (Figure 1). Within the Golgi lumen, the spatial organisation of these enzymes, particularly within distinct Golgi cisternae, directly influences the glycan composition of secreted mAbs (Ferrara et al., 2006). For instance, the compartmentalisation of glycosyltransferases within different cisternae determines the accessibility of glycans to specific enzymes, thereby affecting the maturation and complexity of the *N-*glycan structures. Additionally, the dynamic expression levels of these enzymes, as well as the availability of nucleotide sugar donors influenced by the activity of specific sugar nucleotide transporters in the Golgi membrane, further add layers of complexity to the *N-*glycosylation process. This regulation is mediated by various cellular pathways, including transcriptional and post-transcriptional mechanisms that control the expression of genes encoding these enzymes. Variations in the expression of *N-*glycosylation related genes can lead to significant heterogeneity in glycan profiles, which in turn impacts the biological activity and therapeutic efficacy of mAbs.

Given the complexity of *N-*glycosylation, the challenge lies in identifying the genetic and cellular determinants that drive the production of desired glycoforms. One promising approach is to leverage high-throughput transcriptomic analyses to identify cell lines with more favourable glycan profiles. Transcriptomics provides a comprehensive overview of gene expression patterns, offering the potential to pinpoint key regulatory genes involved in glycan biosynthesis and processing. By correlating gene expression data with glycan profiles, it may be possible to predict genetic determinants that influence the glycosylation outcomes of mAbs. This study aims to explore the relationship between gene expression and glycosylation patterns in mAb production, with the goal of identifying key genetic factors that contribute to desirable glycan profiles. By leveraging a multiomics approach and computational modelling, we seek to identify the regulatory mechanisms that underpin glycan processing and optimise the production of therapeutic mAbs. Understanding these interactions will not only enhance the quality of biopharmaceuticals but also provide valuable insights into the fundamental biology underpinning the glycosylation of mAbs.

## Methods

### Dataset generation

To generate the datasets used in this study, clonal antibody-producing cell lines were cultivated using GSK’s proprietary platform process within the ambr^®^15 miniature bioreactor system. Four different mAbs were considered in this study. Cell samples were collected at key time points during fed-batch production runs, specifically days 0, 6, and 10. Transcriptomic analysis was conducted using bulk RNA sequencing, with RNA counts generated against the Ensembl genome (CriGri_1, GeneModelVersion: 104). For glycan profiling, *N*-glycan analysis was performed using capillary electrophoresis on the GlycanAssure instrument, following the manufacturer’s protocol for glycan release and labelling.

### Multiomic approach for examining relationship between transcription and glycosylation

Experiments were analysed in R (version 4.3.1). Principal component analysis and k-means clustering were completed using the package stats (version 4.3.0) with visualisation of elbow plots requiring the library factoextra (version 1.0.7). The package mixOmics (version 6.24.0) (Rohart et al., 2017) was used to identify predictive genes within clusters. A k-fold cross-validation approach was employed to evaluate the predictive performance of multiomic data integration. Specifically, 10-fold cross-validation was used, splitting the dataset into training and testing subsets for each fold. For each fold, datasets for day 0, day 6, and day 10 transcriptomics and glycan profiling were split into training and testing sets. Sparse Partial Least Squares (sPLS) models were generated for each pair of time points (day 0 vs. day 6, day 0 vs. day 10, day 6 vs. day 10), and correlations between the components were calculated to inform the design of the matrices.

A block-sPLS-DA model was then built using the “block.splsda” function from the mixOmics package, with the design matrix specifying the relationships between the datasets. The number of components was optimised using 10-fold cross-validation with the ‘perf’ function, and the optimal number of features to retain for each dataset was determined using the “tune.block.splsda” function. The final model was trained with the optimal parameters, and predictions were made on the test sets.

The performance of the model was evaluated using the function “auc” on the final models and the function “plotLoadings” allowed for visualisation of the most influential genes within each cluster.

### Computational modelling of glycan processing

A custom computational model developed using Java was previously described (Fisher et al., 2019b; West et al., 2022). Briefly, the model combines the simulation of glycan processing via a Gillespie algorithm based stochastic simulation algorithm (SSA) with iterative parameter adjustment of the used glycosylation reactions via approximate Bayesian computation (ABC) to match experimental data.

The SSA processes 10,000 input glycans through simulated cisternae to generate a glycan profile. The summary statistic used to compare empirical to simulated glycan profiles was calculated using the square difference method, which emphasises dominant glycan species in the profile, at each evaluation of the MCMC chain in the ABC approach.

High performance computing was used to run 30 parallel fitting procedures. The Gelman-Rubin R statistic (Gelman and Rubin, 1992) and Mann-Whitney U tests were used to assess the similarity of the parallel runs and the significance of shifts in parameter distributions, respectively.

## Results

**FIGURE 2:**
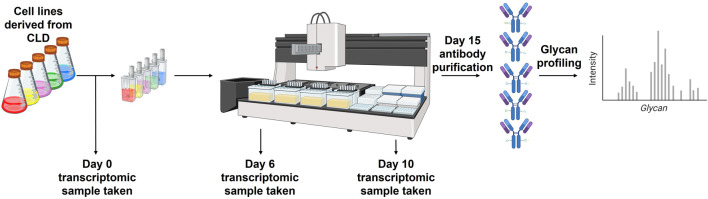
Schematic timeline of mAb production highlighting when sampling was completed.

In this study, we aimed to identify glycosylation-related genes that could serve as predictive markers during CLD for identifying cell lines with the potential to produce mAbs displaying more favourable glycan profiles. Our analysis started with bulk RNA-seq data encompassing the expression levels of 12,935 genes across 53 samples, with each sample measured twice. The sample is representative of 3 distinct time points within the 15 days mAb production process, at which transcriptomic analysis was completed:the cell line at the start of the production run (henceforth referred to as day 0), day 6, and day 10. Each sample is associated with a mAb glycan profile that was collected on day 15, meaning that there is one glycan profile associated with the 3 separate RNA-seq data sets (Figure 2). The full set of data can be separated into two distinct projects, each focusing on a different mAb.

**FIGURE 3:**
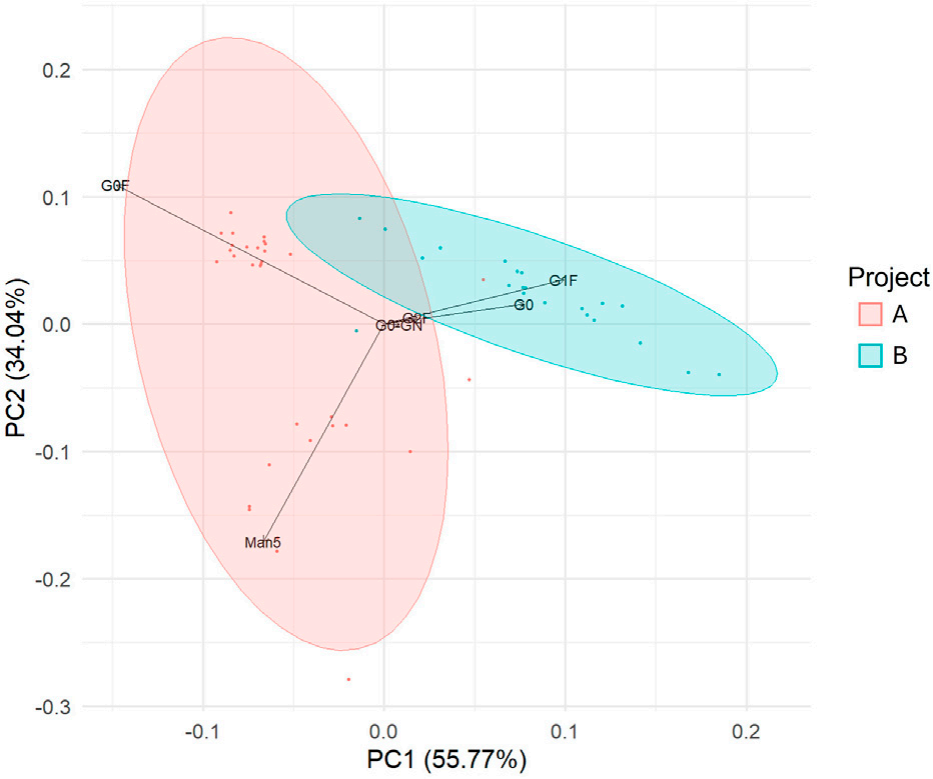
Variance between glycan profiles is predominantly as a result of the project from which the sample originates. Principal component analysis of glycans displayed on the mAbs collected on day 15 of production runs. Each data point corresponds to a single glycan profile, and the samples are coloured by project. n = 53.

Principal component analysis (PCA), conducted on the entire dataset, revealed distinct clustering of samples according to the project of origin, indicating that the variance in *N*-glycan composition was primarily driven by the specific mAb being produced (Figure 3). Given this clear separation, we opted to analyse the two projects independently to ensure that the relationship between gene expression and glycosylation patterns was fully explored within the context of each unique mAb.

**FIGURE 4:**
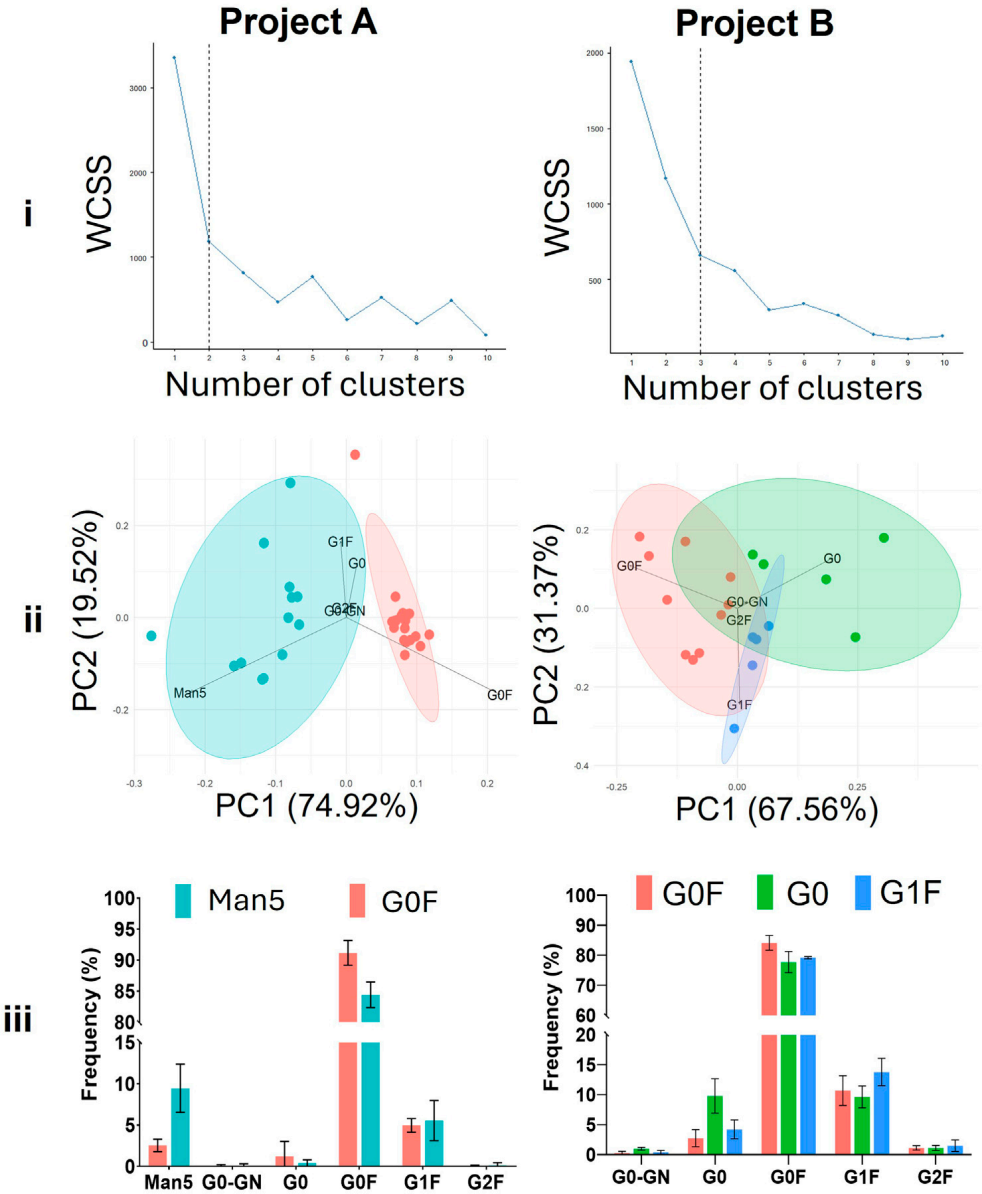
Subdivision of the projects into clusters with different glycoforms using k-means clustering. i) Elbow plots displaying the variance explained as a function of the number of clusters in the data. ii) PCA plot showing the clusters and the different loadings which are prominent within that cluster. iii) Glycan profiles of the clusters depicted in part ‘ii’. WCSS = Within cluster sum of squares.

To investigate intra-project differences in glycan profiles, we applied k-means clustering to the *N*-glycan data from each project. This approach allowed us to categorise the samples into distinct clusters based on their glycosylation patterns, of which one cluster could be more favourable in terms of mAb therapeutic efficacy, as explained below. In project A, the data was best separated into two clusters, while project B required three clusters to achieve a meaningful division (Figure 4i). Overlaying the assigned clusters onto a PCA plot of the glycan profiles for each respective project (Figure 4ii) showed that in project A the two clusters were clearly distinguished, with the separation predominantly driven by the abundance of either Man5 or G0F glycan structures. This observation was supported by the PCA loadings, which highlighted these glycans as key contributors to the variance between clusters, and further confirmed by the quantified glycan profile distributions (Figure 4iii). Between these two clusters the G0F cluster is considered the more favourable therapeutic entity due to enhanced clearance of Man5 containing mAbs (Schlesinger et al., 1978).

For project B, the three distinct clusters were characterised by the abundance of G0, G0F, and G1F glycans (Figure 4ii, 4iii). The G1F cluster is likely the most advantageous, primarily due to the presence of the terminal galactose residue. This galactose residue has been shown to influence key effector functions of mAbs, including enhanced binding to C1q and Fcγ receptors, which in turn boosts complement-dependent cytotoxicity (CDC) and Fcγ receptor activation. Furthermore, G1F glycans contribute to structural stability of the CH2 domain of antibodies, which is crucial for optimal interaction with immune effector molecules (Aoyama et al., 2019). These properties suggest that the G1F cluster in project B may lead to mAbs with superior therapeutic efficacy.

To assess the predictiveness of specific glycosylation-related genes in determining the cluster assignments within each project, we employed mixOmics for multivariate analysis of the RNA-seq data. This analysis focused specifically on genes related to glycosylation to refine our understanding of how these genes contribute to the observed glycan profiles. The analysis was narrowed to relevant genes by compiling a list of 184 glycogenes through a comprehensive literature search (Togayachi et al., 2008). From this initial list, we further refined our focus to 76 genes that are relevant to *N*-linked glycosylation. Using transcriptomic changes in this curated set of 76 genes, we tested their ability to predict cluster assignments within each project using mixOmics. Robustness of the predictive effects was ensured by performing 10-fold cross validation on the project-specific datasets. The data was partitioned into ten sets, where 9 were used for training the model, and the remaining set was used for testing. This process was repeated such that each of the 10 sets served as the test dataset at least once. Genes were considered to have strong predictive power if they were consistently selected in 7 or more of the 10 cross-validation iterations.

**FIGURE 5:**
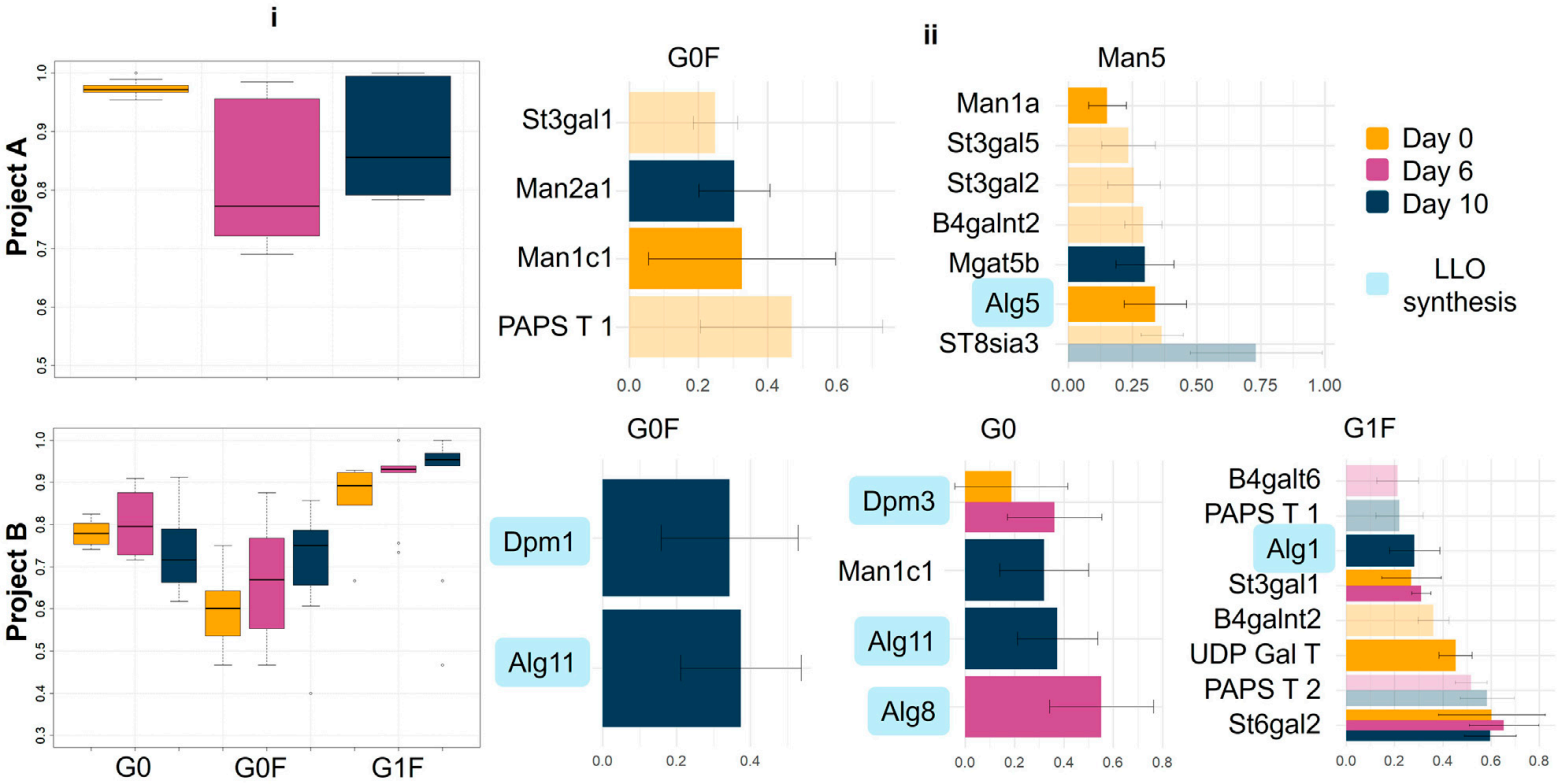
Evaluation and visualization of the final mixOmics models and the genes responsible for predictiveness. i) Area Under the Curve (AUC) plots illustrating the performance of the mixOmics models for predicting glycan profiles. Each plot displays the model’s discriminative ability across different clusters, with higher AUC values indicating better predictive performance ii) Bar plots showing the genes contributing to the model’s predictiveness. The bars represent genes that are elevated in the cluster shown above each plot. The length of each bar corresponds to the gene’s absolute contribution to the prediction, highlighting their relative importance in the model. See Figure 1 for the definition of LLO synthesis.

After completing the 10-fold cross validation, we evaluated the models’ performance using receiver operating characteristic (ROC) curves, which were generated for each of the three sampling days to visualise the discriminative ability of the models. The area under the ROC curve (AUC) was calculated to quantify the accuracy of the classifiers in distinguishing between the different clusters, with an AUC of 1 signifying optimal distinguishing power and a score of 0.5 indicating performance equivalent to random change (Figure 5I). Across both project A and project B, the models generally performed well, with AUC values consistently above 0.5, indicating that they were better than random at distinguishing between the clusters.

However, it is important to note that not all time points performed equally well. In project A, for example, the classifier at day 6 showed a noticeably lower performance compared to day 0 and day 10. This reduced performance at day 6 could be attributed to a high level of intercorrelation between genes at this time point, potentially complicating the model’s ability to accurately differentiate between clusters (Supplementary Figure S1ii). The best predictive power, for project A, was observed at day 0, where the classifier achieved an average AUC value of 0.97. This may have significant implications for CLD, as RNA analysis of a cell line may have predictive power for an optimised glycan profile without the need for a production run.

In contrast, for project B, the AUC values across the three time points were more closely aligned, indicating that the model’s ability to distinguish between clusters remained relatively consistent over time. However, when examining the performance across different clusters, the model was most effective at distinguishing the G1F cluster, as indicated by higher AUC values.

In project A, a total of 33 genes were identified as being predictive of cluster assignments across the three time points (Figure 5ii). z Specifically, 9 genes were predictive at day 0, 21 genes at day 6, and 3 genes at day 10. Given the comparatively lower performance of the model at day 6, the genes identified at this time point were excluded from further evaluation, though they are detailed in Supplementary Figure S2. A corroborative finding in project A is the elevation of Man2a1 levels in the G0F cluster compared to the Man5 cluster. This observation aligns with the known biochemical pathways of glycosylation, where elevated levels of mannosidase II promote the conversion of intermediate glycans (i.e., Man5) into more complex structures, such as G0F. Two different isomers of the earlier acting mannosidase, mannosidase I, showed distinct patterns between the two clusters: Man1c1 was elevated in the G0F cluster, while Man1a was more prevalent in the Man5 cluster. The two isomers act on Man9 glycan precursors by trimming terminal mannose residues until Man5 is formed. The elevated levels of Man1c1 in the G0F cluster are logical, as the faster this trimming occurs, the more time there is for Man5 to be further processed by other enzymes into more complex glycans. Conversely, the elevation of Man1a within the Man5 cluster is unexpected and does not align with the anticipated glycosylation pathway. However, given that Man1a has the smallest contribution to the Man5 component, its influence might be minimal and is currently not a primary focus for analysis. Similarly, the involvement of Mgat5, which is involved in the branching of glycans to make tri- and tetra-antennary glycans, does not have a clear biological explanation in this context.

Alg5, which is involved in synthesis of the oligosaccharide donor used for initiation of *N*-glycan biosynthesis in the ER, does not directly influence glycan processing in the Golgi, and thereby the ratio of different glycoforms (Heesen et al., 1994). Instead, Alg5 will affect the occupancy of glycosylation sites on a range of endogenous proteins within the cell. Increased expression of Alg5 leads to greater site occupancy (Gallo et al., 2022), which in turn increases the competition for glycan processing enzymes in the Golgi, potentially reducing glycan processing on the mAb, thus increasing the proportion of Man5.

In project B (Figure 5ii), several genes associated with the early stages of glycosylation, prior to the action of oligosaccharide transfer to the protein, exhibit elevated levels in the G0 and G0F clusters. These genes, including Dpm1, Alg11, Alg8, and Alg5, are likely contributing to the same effect observed in the Man5 cluster of project A. Higher expression of these genes increases site occupancy on host proteins and thereby competition for the Golgi glycan processing machinery. This may result in the mAb glycans not being processed to their full potential, leading to less complex glycan structures.

The presence of sialyltransferases (St3gal1 and St6gal2) in the G1F cluster could be another indication of competition. Elevated levels of these sialyltransferases may increase their competition with galactosyltransferases for host protein substrates (mAbs are generally not sialylated). This competition could free up galactosyltransferases for the modification of mAb glycans within the G1F cluster, thereby enhancing the galactosylation of these glycans. Finally, increased expression of the UDP-Gal transporter (UDP-Gal T) in the G1F cluster is consistent with our understanding of glycosylation: increased transport of galactose into the Golgi apparatus would provide more substrate for the galactosylation, thus promoting the formation of G1F glycan structures. It is interesting to note though that UDP-Gal T levels are predictive before the start of mAb production on day 0.

**FIGURE 6:**
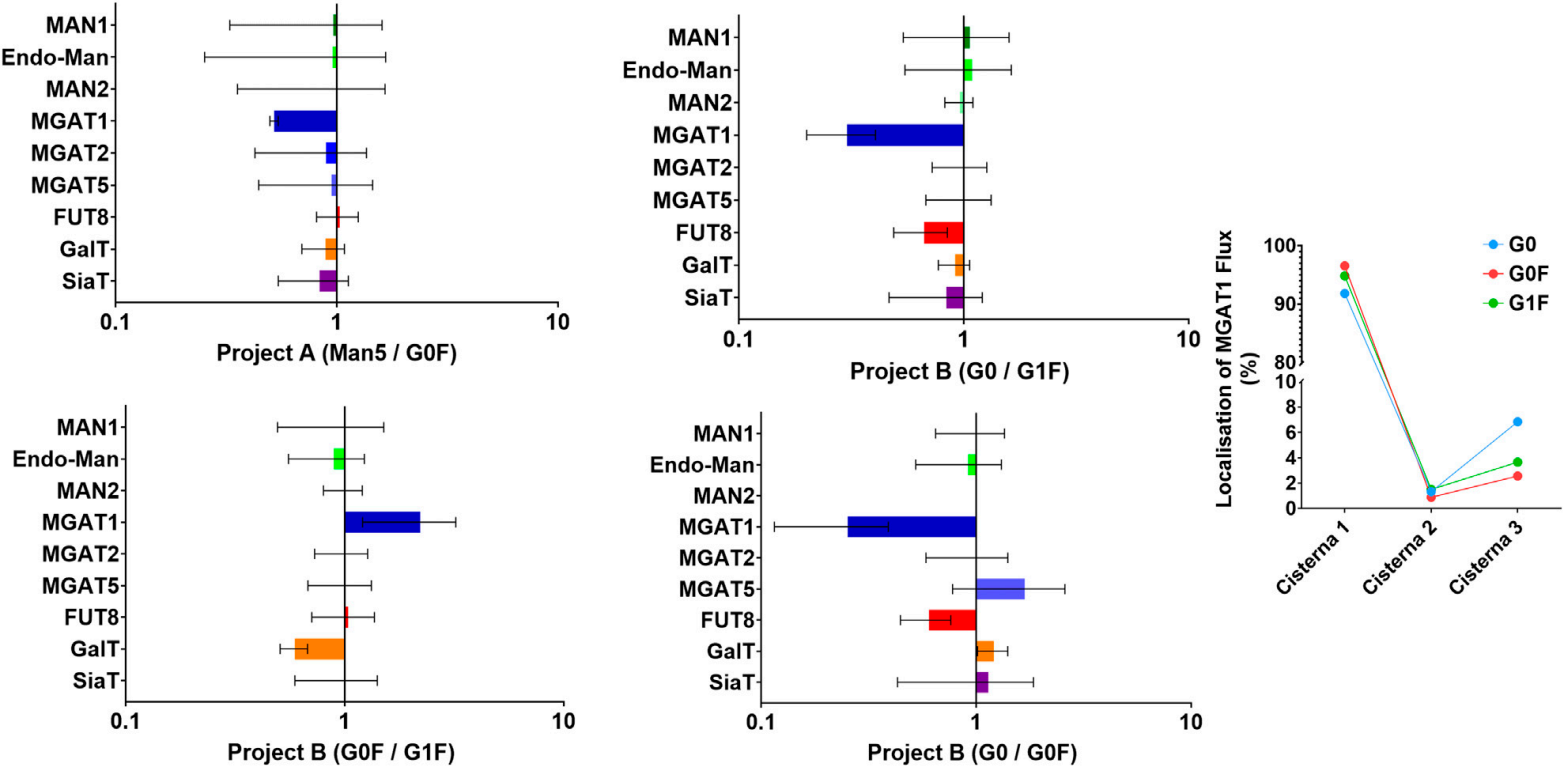
SSA/ABC model predictions illustrating the necessary glycosylation gene changes for achieving different glycan profiles. i) Gene expression changes required to model one cluster’s glycan profile relative to another, for both Project A and Project B. Each plot shows the predicted alterations in glycosylation gene expression needed to shift from one glycan cluster to another, highlighting specific genes and their relative changes ii) Localization of Mgat1 flux in Project B. The plot visualizes the predicted spatial distribution of Mgat1 activity within the Golgi apparatus.

To verify the mechanistic interpretations behind these mixOmics predictions, a stochastic simulation of glycosylation was performed, coupled with approximate Bayesian computation (ABC) fitting to assess changes in levels and localisations of key *N*-glycan processing enzymes across the Golgi apparatus in the different project A and B clusters (Figure 6).

The only significant difference predicted by the computation between the Man5 and the G0F clusters in project A was a reduction in N-acetylglucosaminyltransferase 1 (Mgat1) levels. This reduction in Mgat1 fits well with the observed glycan profile clustering, as lower Mgat1 levels would decrease the conversion of Man5 to more complex glycans. However, it is important to note that this change in Mgat1 expression was not captured by the mixOmics analysis, likely due to the RNA-seq data showing only a slight difference in Mgat1 expression between the clusters (with mean values of 11.5 in the G0F cluster and 12.2 in the Man5 cluster). This suggests that the predicted reduction in Mgat1 activity may not be solely due to differences in RNA expression. The discrepancy between RNA levels and the predicted enzyme activity could be due to several factors. For example, the same RNA levels may lead to varying amounts of Mgat1 protein due to differences in translation efficiency, post-translational modifications, or other Golgi-related factors, such as enzyme localization or substrate availability. Although a change in Mgat1 expression was not picked up by the mixOmics analysis, the decrease in Man2a1 (the immediate next enzyme after Mgat1 in *N*-glycan processing) in the Man5 cluster could represent the same biological effect.

In project B, there are three key comparisons to be made: between the G0 and G0F clusters, the G0 and G1F clusters, and the G0F and G1F clusters. The simulation revealed a consistent reduction in both Mgat1 and Fut8 levels in the G0 cluster compared to the G0F and G1F clusters. The reduction in Mgat1 likely serves to decrease the flux that drives the conversion of G0 glycans, resulting in their retention. The reduction in Fut8 is an obvious prediction that would lead to fewer fucosylated glycans, as observed in the G0 cluster.

The primary difference between the G0F and G1F cluster is, as expected, in the level of galactosylation. The G1F cluster shows higher levels of galactosyltransferase (GalT) compared to the G0F cluster, which is consistent with what is expected. Furthermore, this could be captured in the mixOmics analysis by the increase in the UDP-Gal T as predicted in Figure 4. As the SSA/ABC model only captures overall enzymatic activity, other factors such as substrate availability are subsumed within the activity value. As such, the increased galactosylation activity could be capturing the predicted increase in galactose residues.

Interestingly, the simulation predicted an increase in Mgat1 levels in the G0F cluster compared to the G1F cluster. This increase is coupled with the added complexity that the flux data suggest Mgat1 action to occur earlier in the Golgi with a reduction in activity in later cisternae. On the surface, this pattern seems counterintuitive, as one might expect that increased early flux would allow more time for subsequent galactosylation to occur. However, a deeper look into the role of fucosylation provides a plausible explanation.

Fucosylation can compete with galactosylation and can inhibit it when both processes target the same glycan. In the G0F cluster, the observed shift of Mgat1 activity to an earlier stage may be a compensatory mechanism to facilitate fucosylation in the absence of significant competition. By enabling Mgat1 to act earlier, the glycan structure is primed for fucosylation sooner, maintaining the window of opportunity for galactosylation to occur later in the Golgi. However, it’s important to include a disclaimer here. Fut8 operates at a slower rate compared to other glycosylation enzymes. This slower reaction rate needed to be accurately represented in the simulation, but since enzyme activity and concentration are intertwined in our model, a workaround was implemented. We introduced an artificial enzymatic process, equivalent to a hidden Markov state that temporarily restricts access to the glycan until fucosylation is complete. While this approach successfully decouples fucosylation’s slow rate from other reactions, it also introduces a potential artefact where unprocessed glycans may be retained across the Golgi longer than expected, resulting in fewer overall modifications.

While the simulation suggests that Mgat1 acts earlier in the G0F cluster to facilitate fucosylation and limit galactosylation, this result should be interpreted cautiously. The key takeaway is that fucosylation is likely competing with galactosylation. This dynamic helps to explain the less elaborated glycan structures observed in the G0F cluster. The interaction between fucosylation and galactosylation, rather than Mgat1 alone, is likely driving these differences.

## Discussion

Our study identified glycosylation related genes as predictive markers for cell lines that produce mAbs with favourable glycan profiles. By correlating gene expression patterns with glycan profiles, we provided insights into potential genetic determinants of glycan heterogeneity. However, our findings also highlight a critical limitation: the link between transcriptomic data and glycosylation outcomes is not a straightforward relationship.

The predictive relationship between transcriptomics and glycomics observed in our study aligns with findings from previous research, such as the work by Nairn et al. (2012). Their study revealed correlations between transcript expression and glycan abundance in various animal tissues, suggesting that transcript levels of glycosylation related genes could influence the overall glycan profile (Nairn et al., 2012). In our study, we similarly observed that distinct clusters of glycan profiles could be predicted from transcriptomic data. However, as Nairn et al. noted, not all glycan structures correspond directly to transcript levels of the biosynthetic enzymes responsible for their production. This implies that other regulatory mechanisms are also at play. This is particularly obvious when we examine the insights gained from the SSA/ABC modelling. One key finding was the role of Mgat1 in determining the differences between favourable and unfavourable glycan profiles. The modelling demonstrated that it was not just the activity level of Mgat1, which may be influenced by transcriptomic levels, but rather it’s localisation across the Golgi cisternae that played a pivotal role in influencing glycan profiles (Bailey Blackburn et al., 2016; Fisher et al., 2019a). This observation emphasises the importance of considering enzyme localisation, something which cannot currently be predicted from transcriptomic data. It may be possible to gain further mechanistic insight with a larger set of genes which also includes trafficking machinery, but this is currently unavailable and certainly beyond the scope of this study.

Our study has identified differential responses at the protein-specific level which are consistent with the hierarchical nature of *N*-glycosylation described by Arigoni-Affolter et al. (2024). While cellular *N*-glycome changes could be correlated with glycosylation enzyme expression, individual mAbs exhibited unique glycosylation patterns that were likely influenced in concert by their specific protein structure with the local glycosylation environment. This is another level of specificity that cannot be captured by the transcriptomic data and is currently also very hard to model using the SSA/ABC model. Importantly, in the case of therapeutic mAbs the well-known site-specific processing of the attached *N*-glycans is significantly different from the “average” endogenous processing requirements in the Golgi (Rudd and Dwek, 1997). Therefore, changes in endogenous glycan processing reactions that would normally not act on mAbs can have unforeseen consequences on mAb glycan processing due to altered competition with enzymes that do act on mAbs.

Moreover, despite the strong correlations and predictiveness observed between transcriptomics and glycan profiles, several caveats must be considered. A major limitation of using transcriptomic data to predict glycan outcomes is the realisation that changes in mRNA levels do not always translate directly into changes in protein levels. Protein levels within the cell are governed by a balance of synthesis and degradation processes, with transcriptomics only capturing the first half of this lifecycle (Liu et al., 2016). This disconnect may lead to discrepancies between predicted and actual glycosylation patterns, as enzyme activity, and not solely protein abundance, is influenced by factors such as intracellular localisation within the Golgi cisternae and post-translational modifications. Additionally, enzyme interactions, relative abundances, and substrate competition play critical roles in determining flux through various branches of the *N*-glycosylation pathway. For instance, while our analyses focus on *N*-glycosylation, it is worth noting that key substrates, like UDP-Gal, are involved in multiple glycosylation pathways, including O-glycosylation and glycolipid synthesis. The production of a single protein at high levels, such as when producing a therapeutic mAb, may alter cellular physiology and impact flux through these other glycosylation pathways. The inherent complexity and interdependence of the processes present challenges in predicting glycan outcomes based solely on transcriptomic data. However, despite these challenges, the insights gained from our analyses lay the groundwork for more refined predictive models.

Nonetheless, the findings from our study could have significant implications for CLD, particularly in optimising cell lines early on in the production timeline. A noteworthy observation from project A was that the highest predictive power was achieved prior to beginning the production run. This early time point’s strong predictive capability suggests that critical determinants of glycan heterogeneity may be established early in the culture process. For CLD, this means that early transcriptomic profiling could be a powerful tool for selecting cell lines likely to yield desirable glycosylation patterns later in the production process. In particular, higher levels of Alg5 and the UDP-Gal transporter in candidate cell lines are indicative of being able to produce mAbs with higher therapeutic efficacy.

One of the key genes identified at this early time point was Alg5. Although Alg5 does not directly influence glycan processing in the Golgi apparatus, it’s prominent influence on glycosylation could result from its ability to increase occupation of glycosylation sites on a range of endogenous proteins within the cell (Gallo et al., 2022). This increased site occupancy can create competition for glycan processing enzymes in the Golgi, thereby reducing the processing of glycans on mAbs. For CLD, this suggests that monitoring and modulating Alg5 expression early in the cell culture process could be critical in controlling the glycan profile of mAbs. Specifically, limiting Alg5 expression might reduce competition for glycosylation enzymes, thereby enhancing the processing of mAb glycans into more complex and therapeutically favourable forms.

In addition to Alg5, UDP-Gal T also emerged as a significant predictive marker in project B. Specifically, UDP-Gal T was notably predictive for the G1F cluster prior to beginning the production run. High expression of UDP-Gal T facilitates the addition of galactose, which is essential for generating more G1F glycans. Therefore, monitoring and optimising UDP-Gal T expression early in the culture process could further improve glycan profiles by promoting the formation of complex, galactosylated glycans that are advantageous for therapeutic efficacy.

Future work should focus on integrating proteomic data in addition to the transcriptomics used here to provide a more comprehensive view of factors influencing glycan heterogeneity. Additionally, exploring the subcellular localisation of glycosylation enzymes and their interactions with specific glycoproteins could offer deeper insights into the mechanisms driving glycosylation patterns. Understanding these processes will not only enhance the production of therapeutic mAbs with desirable glycan profiles but also contribute to the broader field of glycoscience, providing valuable knowledge on the regulation of protein glycosylation in eukaryotic cells.

## Data Availability

The data analysed in this study is subject to the following licenses/restrictions: We are describing the use of a dataset generated by GSK to make predictions of useful features during cell line development in biopharmaceutical production. The dataset we used was provided by GSK with a confidentiality clause, and while there are co-authors from the company on the manuscript, the company would not want to make the dataset openly available. The datasets used in the article are not available because they relate to GSK confidential information. No requests to access the datasets will be granted. For clarifications on this statement reach out to pavlos.x.kotidis@gsk.com.
