# Supplementary material for: Transcriptomics as a predictor of biopharmaceutically favourable glycan profiles
Source: Front Cell Dev Biol. 2024 Dec 17;12:1504381. doi: 10.3389/fcell.2024.1504381 (PMC11686447; doi:10.3389/fcell.2024.1504381)

Supplementary Figure 1 Correlation between gene expression levels across different time points in project A. i) Correlation on day 0, ii) day 6, iii) day 10. Correlation is indicated by the colour of the dot with dark blue being equal to a correlation coefficient of 1 and dark red -1.

i)

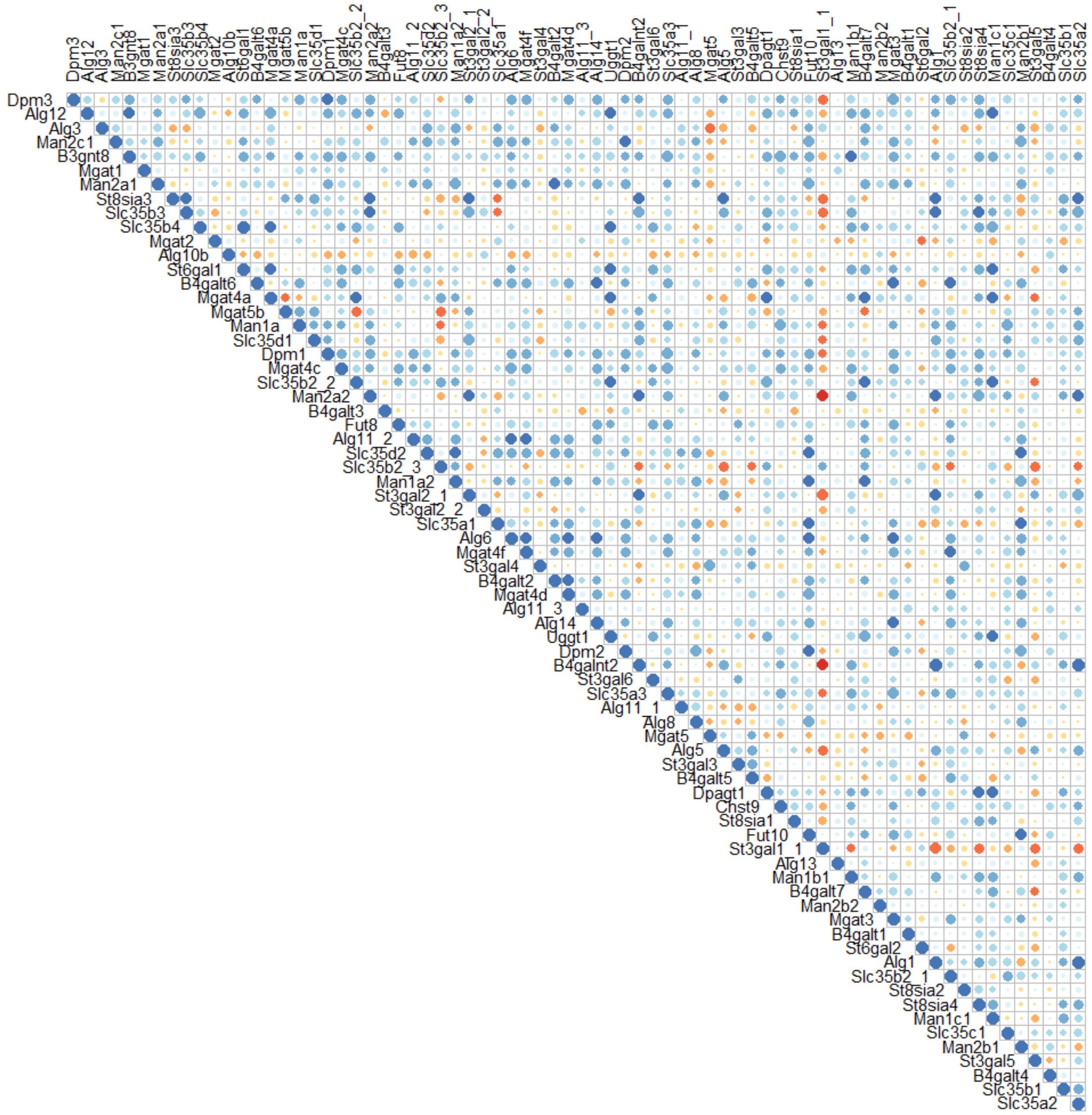

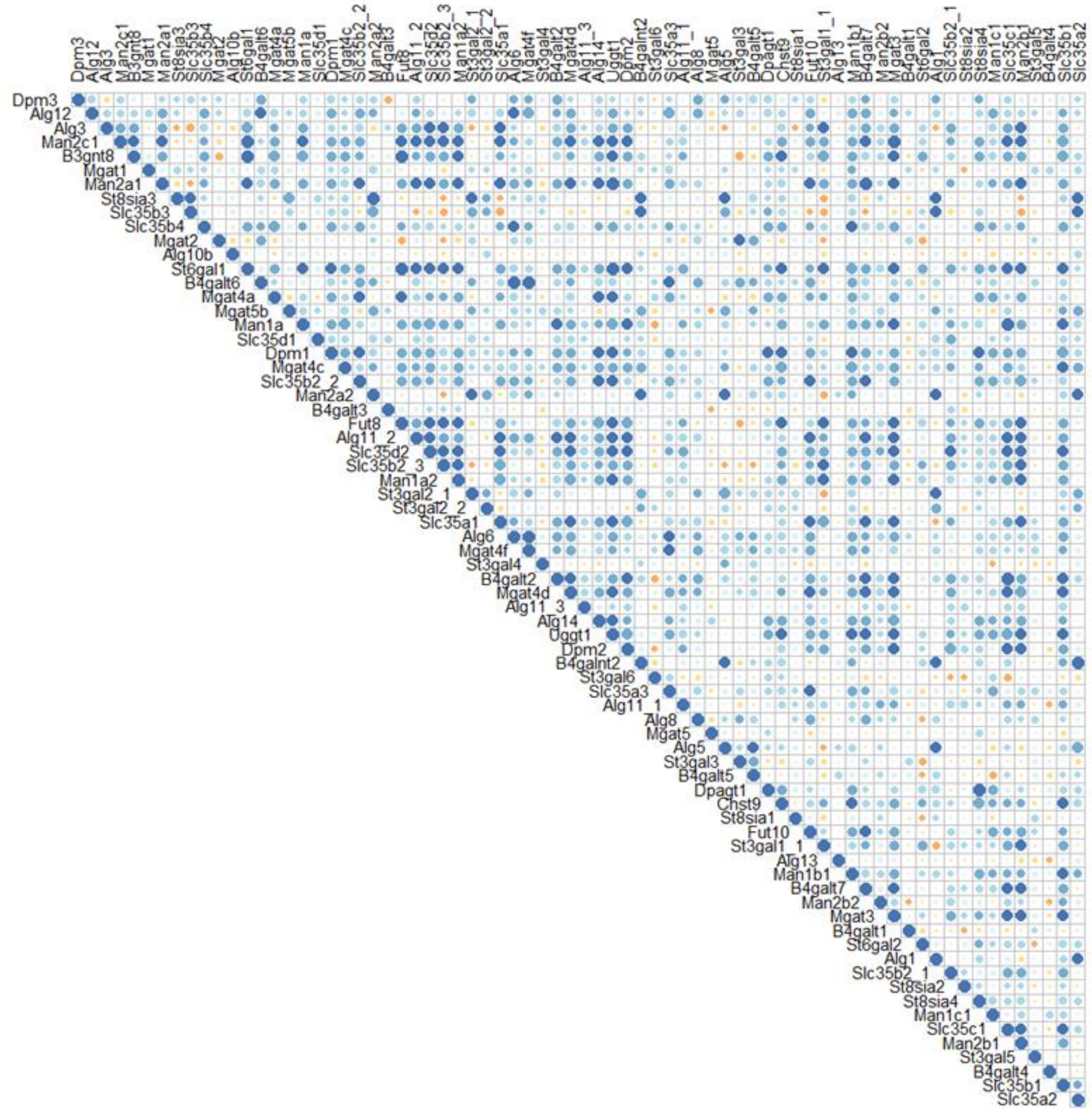
Dpm3
Alg12
Alg3
Man2c1
B3gnt8
Mgat1
Man2a1
St8sia3
Slc35b3
Slc35b4
Mgat2
Alg10b
St6gal1
B4galt6
Mgat4a
Mgat5b
Man1a
Slc35d1
Dpm1
Mgat4c
Slc35b2_2
Man2a2
B4galt3
Fut8
Alg11_2
Slc35d2
Slc35b2_3
Man1a2
St3gal2_1
St3gal2_2
Slc35a1
Alg6
Mgat4f
St3gal4
B4galt2
Mgat4d
Alg11_3
Alg14
Uggt1
Dpm2
B4galnt2
St3gal6
Slc35a3
Alg11_1
Alg8
Mgat5
Alg5
St3gal3
B4galt5
Dpagt1
Chst9
St8sia1
Fut10
St3gal1_1
Alg13
Man1b1
B4galt7
Man2b2
Mgat3
B4galt1
St6gal2
Alg1
Slc35b2_1
St8sia2
St8sia4
Man1c1
Slc35c1
Man2b1
St3gal5
B4galt4
Slc35b1
Slc35a2

ii)

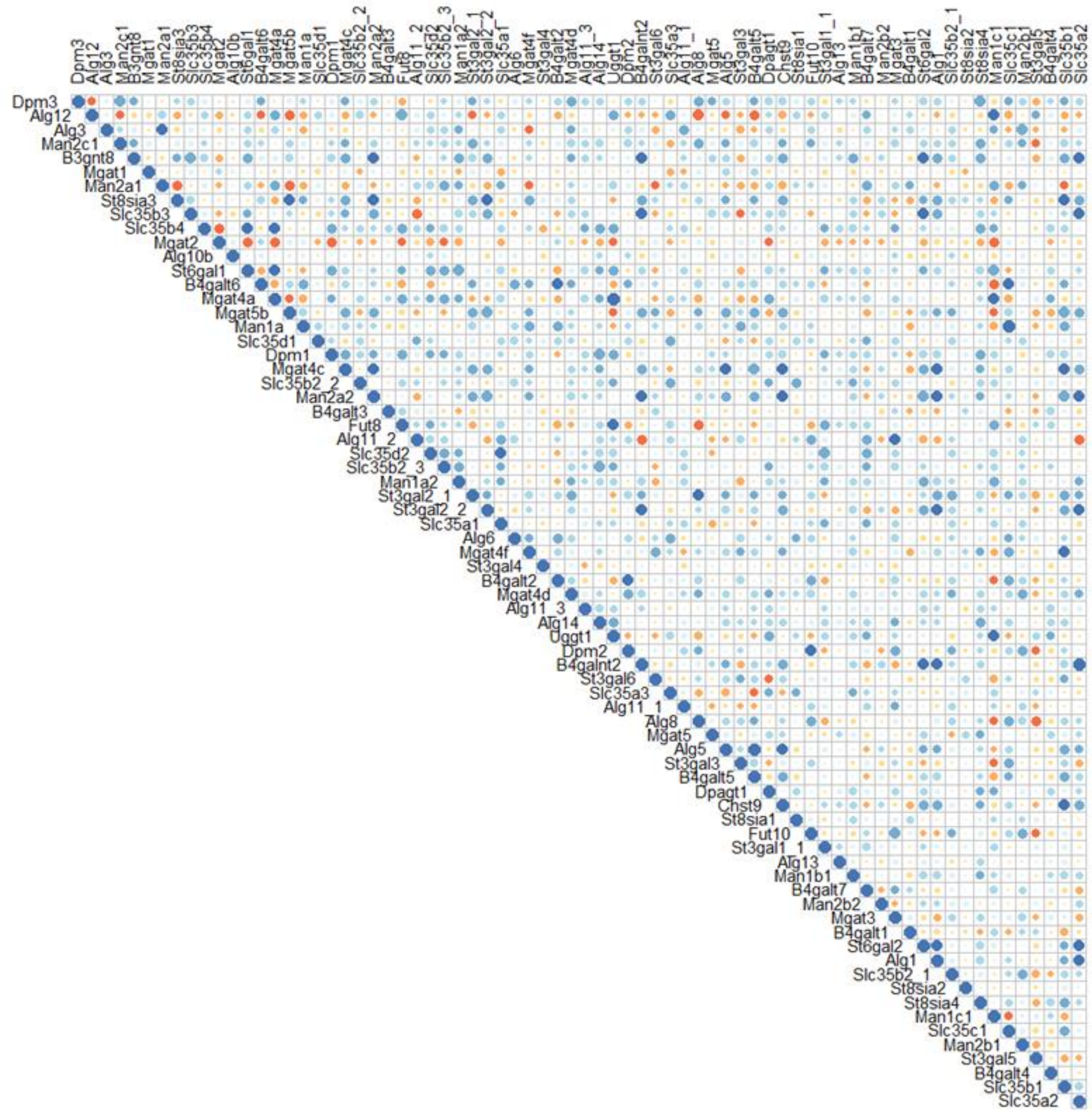
Dpm3
Alg12
Alg3
Man2c1
B3gnt8
Mgat1
Man2a1
St8sia3
Slc35b3
Slc35b4
Mgat2
Alg10b
St6gal1
B4galt6
Mgat4a
Mgat5b
Man1a
Slc35d1
Dpm1
Mgat4c
Slc35b2_2
Man2a2
B4galt3
Fut8
Alg11_2
Slc35d2
Slc35b2_3
Man1a2
St3gal2_1
St3gal2_2
Slc35a1
Alg6
Mgat4f
St3gal4
B4galt2
Mgat4d
Alg11_3
Alg14
Uggt1
Dpm2
B4galnt2
St3gal6
Slc35a3
Alg11_1
Alg8
Mgat5
Alg5
St3gal3
B4galt5
Dpagt1
Chst9
St8sia1
Fut10
St3gal1_1
Alg13
Man1b1
B4galt7
Man2b2
Mgat3
B4galt1
St6gal2
Alg1
Slc35b2_1
St8sia2
St8sia4
Man1c1
Slc35c1
Man2b1
St3gal5
B4galt4
Slc35b1
Slc35a2

iii)

Supplementary Figure 2 Evaluation and visualisation of mixOmics model for day 6 in project A. Bar plots showing the genes contributing to the model's predictiveness. The bars represent genes that are elevated in the cluster shown above each plot. The length of each bar corresponds to the gene's absolute contribution to the prediction, highlighting their relative importance in the model.

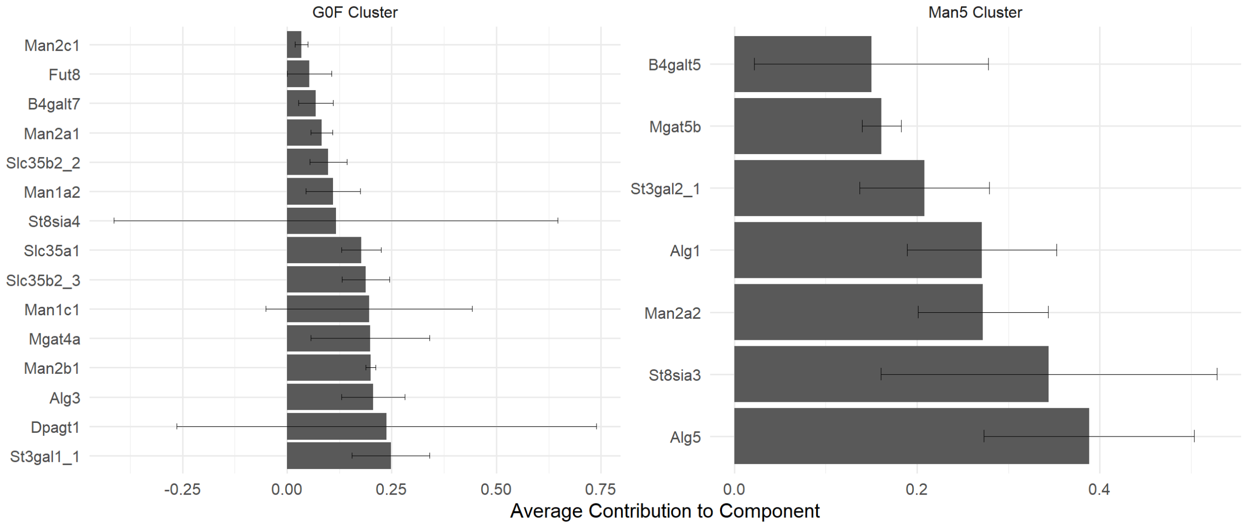

Supplement: Supplementary file 1 [file DataSheet1.pdf]
